# Atomic-scale thermopower in charge density wave states

**DOI:** 10.1038/s41467-022-32226-y

**Published:** 2022-08-03

**Authors:** Dohyun Kim, Eui-Cheol Shin, Yongjoon Lee, Young Hee Lee, Mali Zhao, Yong-Hyun Kim, Heejun Yang

**Affiliations:** 1 Department of Energy Science, Sungkyunkwan University, Suwon, Korea; 2 Department of Physics, Korea Advanced Institute of Science and Technology (KAIST), Daejeon, Korea; 3 Center for Integrated Nanostructure Physics (CINAP), Institute for Basic Science, Suwon, Korea; 4 Interdisciplinary Materials Research Center, College of Materials Science and Engineering, Tongji University, Shanghai, People’s Republic of China

**Keywords:** Thermoelectrics, Electronic properties and materials

## Abstract

The microscopic origins of thermopower have been investigated to design efficient thermoelectric devices, but strongly correlated quantum states such as charge density waves and Mott insulating phase remain to be explored for atomic-scale thermopower engineering. Here, we report on thermopower and phonon puddles in the charge density wave states in 1T-TaS_2_, probed by scanning thermoelectric microscopy. The Star-of-David clusters of atoms in 1T-TaS_2_ exhibit counterintuitive variations in thermopower with broken three-fold symmetry at the atomic scale, originating from the localized nature of valence electrons and their interlayer coupling in the Mott insulating charge density waves phase of 1T-TaS_2_. Additionally, phonon puddles are observed with a spatial range shorter than the conventional mean free path of phonons, revealing the phonon propagation and scattering in the subsurface structures of 1T-TaS_2_.

## Abstract (web summary)

Microscopic origins of thermopower are investigated to design efficient thermoelectric devices. Here, the authors report thermopower and phonon puddles in the charge density wave states in 1T-TaS_2_ by scanning thermoelectric microscopy.

## Introduction

Quantum phases in strongly correlated electronic systems have provided unprecedented opportunities in energy device applications^1^_._ For example, Mott insulators, spin density waves, and charge density waves (CDW) have been explored for practical electronic and spintronic devices with ultralow energy operation^2–7^. Among the various energy device applications, efficient thermoelectricity has been demonstrated in correlated quantum phases^8–13^. However, while emerging spintronic and electronic devices based on correlated electrons have been deeply explored, thermoelectric applications have as yet only been investigated at the device scale, and the microscopic origins of the thermopower in quantum phases remain to be explored.

It has been reported that a narrow distribution of electron energy states can maximize thermopower^14^. Various quantum states, such as the Mott insulating gap and CDW states, possess singular electronic band structures with a narrow bandgap opening near the Fermi level, and strong electron-electron and electron-phonon interactions. For these reasons, CDW phases have been considered promising for practical thermoelectric devices, which has motivated extensive studies, particularly with layered materials. For example, engineering the transition temperature of CDW phases has been achieved by thickness control and electric gating in layered transition metal dichalcogenides^15–18^. However, the role of strongly correlated electrons and their coupling with phonons has not yet been explored, particularly with respect to unique thermopower generation in CDW materials, where electron-electron and electron-phonon interactions critically matter for energy generation over interatomic distances.

In solid-state materials, temperature is not considered a physical quantity that varies at the atomic scale. Local temperature variation is related to local thermal conductance where the propagation and scattering of electrons and phonons play a critical role with a spatial range of their mean free paths^19–21^. Transient lattice temperatures produced by laser illumination have recently been used for nonthermal CDW states, but the spatial dimension of the lattice temperature remains similar to that of the phonon dynamics, reaching tens of micrometers^22^. The phonon dynamics of local temperature variation has not been demonstrated at the nanometer scale. It has been reported that scanning thermoelectric microscopy (SThEM) can be used to probe atomic-scale thermopower and temperature gaps. Nevertheless, previous studies have focused on local Seebeck coefficients determined by weakly correlating electrons without thoroughly appreciating the phonon dynamics in the material^23–27^.

Here, we report on the atomic-scale investigation of thermopower and phonon puddles in the CDW states of 1T-TaS_2_ using SThEM. In previous studies, large thermopower has been observed at the device scale in the commensurate CDW phase in 1T-TaS_2_, which is promising for space applications with radiation hardness^28,29^. In our study, the Star-of-David (SoD) clusters of tantalum and sulfur atoms exhibit drastic and counterintuitive variations in thermopower (4 mV) over a distance of 1 nm. Moreover, in contrast with former scanning tunneling microscopy (STM) studies^30,31^, the spatial variation of thermopower in 1T-TaS_2_ exhibits a broken three-fold rotational symmetry, which can be explained by the unique interlayer coupling in the layered material. The localized electrons with large effective mass^32^, which generate the Mott insulating gap in the commensurate CDW phase, contribute to the atomic-scale thermopower features in 1T-TaS_2_. In addition, local heat transfer rates with a spatial range shorter than the conventional mean free path of phonons, called phonon puddles in our study, were probed for the first time in 1T-TaS_2_ with SThEM. The phonon puddles reflect the propagation and scattering of phonons in the subsurface structures of 1T-TaS_2_, which determines local heat dissipation in the CDW phase.

## Results

### Thermopower from the nearly commensurate CDW phase of 1T-TaS_2_

**Fig. 1:**
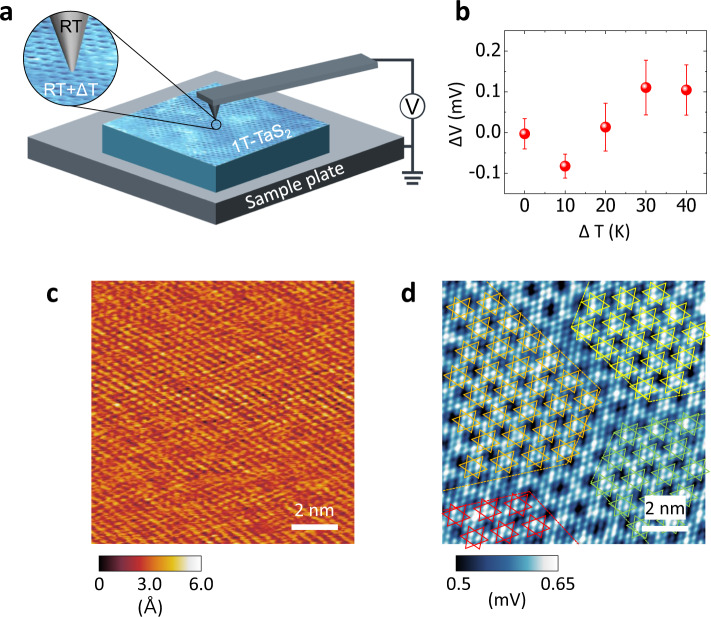
SThEM of the nearly commensurate CDW phase in 1T-TaS_2_. **a** Schematic of the SThEM with 1T-TaS_2_. The temperature of the sample can be controlled from T=78 K to 450 K, and the tip is kept at room temperature in the SThEM. **b** Averaged thermopower (ΔV) as a function of temperature difference (ΔT). The ΔT is defined by T_sample_-T_tip_. Error bars represent standard deviations. **c**, **d** Topography **c** and SThEM images **d** of the nearly commensurate CDW phase in 1T-TaS_2_ with ΔT = 30 K. Sulfur atoms and the SoD clusters are observed in both images. Besides the fine structures, four domains are visible in **d**. The sulfur atoms in the SoD are overlain to highlight the domain boundaries.

A bulk crystalline 1T-TaS_2_ sample was cleaned in-situ by exfoliating the top several layers in an ultrahigh vacuum (UHV) chamber, and then transferred to the SThEM head in the same chamber without being exposed to air. A schematic of the SThEM in our study is illustrated in Fig. 1a. The platinum-coated metallic cantilever (tip) was kept at room temperature while the electrically grounded 1T-TaS_2_ sample was heated or cooled by liquid nitrogen to generate a nominal temperature gradient (ΔT) for thermopower generation. The tip and sample were thermally separated in our SThEM, where the ΔT-induced thermoelectric voltage (ΔV) at the tip-sample interface was measured by a high-impedance electrometer in contact-mode. In addition to the thermopower mapping in SThEM, STM and tunneling spectroscopy (i.e., dI/dV) were also conducted using a separated preamplifier, which allowed the combined and in-situ study of local electronic band structures and corresponding thermoelectricity at the atomic scale.

The thermopower of 1T-TaS_2_ around room temperature is shown as a function of ΔT in Fig. 1b. Each point in Fig. 1b is an averaged value of thermoelectric voltages mapped at a given ΔT. The overall thermoelectric voltages are smaller than 0.1 mV, indicating the small Seebeck coefficient (<10 µV/K) of 1T-TaS_2_ of the nearly commensurate CDW phase above room temperature (T = 300 K). We note that a singular electronic band structure such as a Mott insulating gap, which was required for high thermopower in previous studies, is not present in the nearly commensurate CDW phase. The sign reversal of ΔV appears around ΔT = 10 K, which is consistent with a previous report^8^, indicating the 1T-TaS_2_ has a semimetallic nature, where both electrons and holes contribute to the thermopower in the temperature range.

Atomic force microscopy to observe topography and SThEM were simultaneously conducted in our study. A series of topographic and thermopower images obtained at ΔT = 30 K (i.e., T_tip_ = 300 K and T_sample_ = 330 K) are shown in Fig. 1c, d. The detailed conditions used for scanning are listed in the methods. The sulfur atoms on the top surface of 1T-TaS_2_ form a hexagonal lattice structure in the topography, with structural ordering of the nearly commensurate CDW phase: SoD clusters of atoms with domain structures. The lattice distortion is expressed by a superlattice of $\sqrt{13}$ × $\sqrt{13}$ with a rotation angle of 12° in the topography, which is consistent with former studies on the nearly commensurate CDW phase in 1T-TaS_2_^33^.

The thermopower image at ΔT = 30 K in Fig. 1d more clearly shows the structure of the nearly commensurate CDW phase with the apparent SoD feature at atomic-scale, with an averaged Seebeck coefficient of 8 µV/K. The spatial modulation of ΔV at ΔT = 30 K follows the atomic arrangement of sulfur in the SoD clusters of the CDW of 1T-TaS_2_ with a ΔV variation of 0.1 mV (marked in the scale bar).

We employed both topography and SThEM to probe the top sulfur atoms of a heated sample of 1T-TaS_2_. From the results, we interpret that the ΔV(r) mapping might be dominated by atomic-scale variation in thermopower. The SoD is overlain to highlight the four domains in the nearly commensurate CDW phase, shown by different colors in Fig. 1d. The yellow, orange, red, and green stars represent the SoD of 1T-TaS_2_ in individual domains. The small commensurate CDW areas are explained by the fact that the temperature of the sample (T_sample_ = 330 K) is close to the transition temperature of the incommensurate CDW phase in 1T-TaS_2_.

### Stacking order-dependent thermopower from the commensurate CDW phase of 1T-TaS_2_

**Fig. 2:**
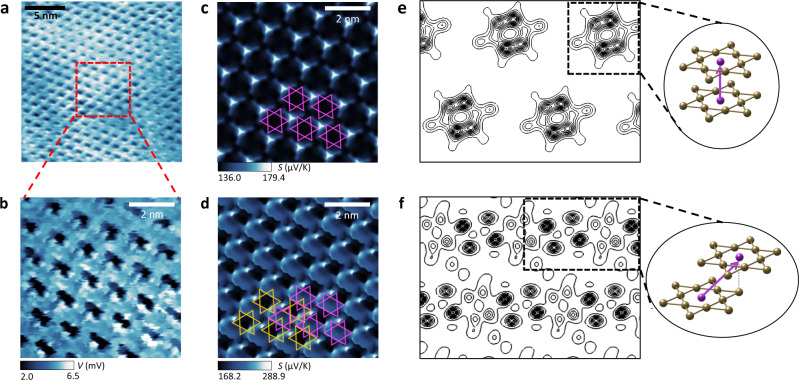
SThEM of the Mott insulating and commensurate CDW phase in 1T-TaS_2_ with two stacking orders. **a** A SThEM image of the commensurate phase of 1T-TaS_2_ at T_sample _= 78 K. The Mott insulating gap and p-type nature of thermoelectricity appear as large and positive thermoelectric voltage signals. The ΔV varies up to 4 mV across the SoD clusters of atoms. **b** A magnified image of the SThEM of the commensurate CDW phase in 1T-TaS_2_, showing large ΔV fluctuation. **c**, **d** Theoretical scanning Seebeck profiles of *A* stacking **c** and *L* stacking **d** bilayer commensurate CDW phase in 1T-TaS_2_. The magenta and orange SoD in **d** denote the top and bottom layer CDW, respectively. **e**, **f** The valence electron densities of *A* stacking **e** and *L* stacking **f** bilayer in the middle of the two layers are shown with their atomic configuration for stacking. Only Ta atoms are displayed for clarity.

The thermopower from the commensurate CDW phase in 1T-TaS_2_, involving a Mott insulating gap, was investigated by SThEM at T_sample_ = 78 K (i.e., ΔT = 220 K with T_tip_ = 300 K). The thermopower image of 1T-TaS_2_ at ΔT = 220 K shows an averaged ΔV = 4.7 mV in Fig. 2a. The polarity of the thermoelectric voltage is consistent with the hole carrier-dominant thermoelectric characteristics reported in the device studies of 1T-TaS_2_^9^.

In contrast to the thermopower image at T_sample_ = 330 K (i.e., in the nearly commensurate CDW phase), two distinct features were newly observed in the thermopower mapping at T_sample_ = 78 K; (1) the three-fold rotational symmetry of SoD is broken, and (2) a large modulation amplitude of thermopower (ΔV up to 4 mV) occurs over a distance shorter than 1 nm as shown in Fig. 2a, b.

It was possible to explain the broken symmetry and drastic thermopower variation over a spatial range of the SoD using first-principles calculations (see Supplementary Materials). It has been reported that two stacking orders are present in 1T-TaS_2_: *A* and *L* stackings^30,34,35^. Our calculations show that the two stacking orders generate distinct Mott insulating gaps of 230 meV (for *A* stacking) and 85 meV (for *L* stacking) with different rotational symmetry in thermopower in 1T-TaS_2_ (Fig. 2c, d). While a single atomic layer of 1T-TaS_2_ without any stacking order cannot reproduce the thermoelectric imaging with the broken symmetry (Supplementary Fig. 7), multiple layers of 1T-TaS_2_ with a certain stacking order (i.e., *L* stacking^30^) demonstrate the two key features of thermopower (broken three-fold symmetry and drastic thermopower changes) as shown in Fig. 2b, d.

The interlayer coupling with the *L* stacking order is critical to determining the broken symmetry and exceptional thermopower variation (120 µV/K) at the atomic scale (1 nm) in our calculations. The sliced valence electron densities of the *A* and *L* stacking bilayer 1T-TaS_2_ (in the middle of the two layers) are shown in Fig. 2e, f with their atomic configurations. The *L* stacking bilayer exhibits significant anisotropic and diagonal interlayer hybridization, with antibonding nature between the *p*_z_ orbitals of the sulfur atoms, contributing to the broken symmetry and drastic thermopower variation.

The other representative stacking order (the *A* stacking) induces a low spatial variation in thermopower (43.3 µV/K) with three-fold rotational symmetry (Fig. 2c), similar to the single-layer case. We note that former STM studies could not probe the atomic morphology and electronic band structures with different stacking orders^31,36^, but the present SThEM could. Further details about the local electronic structures and thermopower with other stacking orders are summarized in the Supplementary Materials (Figs. S8–S10).

### Phonon puddles with the Mott insulating gap in 1T-TaS_2_

**Fig. 3:**
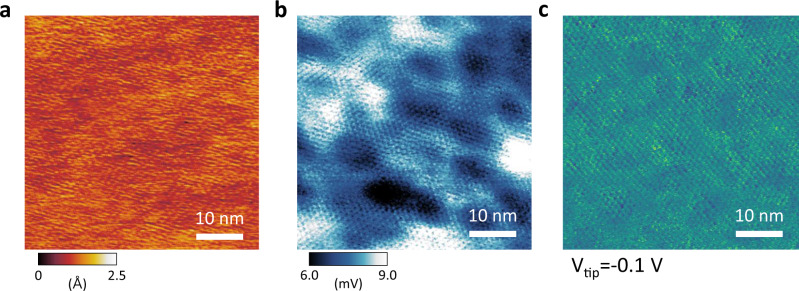
Thermopower and charge puddles in the commensurate CDW phase in 1T-TaS_2_ by SThEM and STM. **a**, **b** Topography **a** and thermopower **b** images over a distance of 100 nm at T_sample_= 78 K. Despite the flat nature of the topography in **a**, the thermopower fluctuation shows a spatial variation of up to 3 mV, which is distinct from charge puddles in 1T-TaS_2_. The bulk single crystal of our 1T-TaS_2_ excludes the possibility of substrate effects for the ΔV fluctuation. **c** A dI/dV mapping image by STM with a tip voltage bias of V_tip_=−0.1 V. Charge puddles near the Fermi level are observed with a subtle contrast, which suggests an additional origin for the large ΔV fluctuation in **b**.

The concept of puddles has been used in STM and SThEM studies to indicate inhomogeneous charge and thermopower distributions (charge and thermoelectric puddles)^25^. Large-scale thermopower puddles were observed by SThEM on the atomically-flat surface of the commensurate CDW phase in 1T-TaS_2_, as shown in Fig. 3a, b. The thermopower variation (Fig. 3b) is not coupled to the flat topographic feature (Fig. 3a) of 1T-TaS_2_ in the spatial range. While the thermopower puddles were observed in most areas in the commensurate CDW phase in 1T-TaS_2_, the nearly commensurate CDW phase (Fig. 1 and S3) near room temperature did not exhibit the large-scale thermopower fluctuation with a spatial dimension of 20 nm. Thus, we interpret the exotic thermopower puddles to be a consequence of the Mott insulating gap in the commensurate CDW; the domains and their boundaries shown in Fig. 1 are not the origin of the thermopower puddles in Fig. 3b.

According to our first-principles calculations, the local Seebeck coefficient (i.e., thermopower) is critically changed by the localized electronic states or the Fermi level in the Mott insulating phase of 1T-TaS_2_ (Supplementary Fig. 7). The strong electron-electron interaction that opens the Mott insulating gap reduces the electron density and weakens electronic screening in the correlated phase, which increases spatial thermopower variation from subsurface impurities, strain, or defects over tens of nanometer range. The absence of strongly correlated electrons for the Mott insulating gap also explains the absence of ΔV fluctuation in the nearly commensurate CDW phase, where effective electronic screening can occur due to the abundant electrons at the Fermi level of the 1T-TaS_2_. We note that local fluctuations in electronic structures have not been observed in previous studies based on 1T-TaS_2_ using STM^37–39^.

We conducted STM and tunneling spectroscopy (dI/dV) mapping to investigate the contribution of the local variation in electronic structure to the thermopower puddles seen in Fig. 3b. It has been reported that local band structures produce charge puddles in graphene^25^. In graphene, the weak screening of external impurities produces the spatially-varying charge carrier density (i.e., charge puddles), which can be observed by STM^35^. The dI/dV mapping image in Fig. 3c hardly shows charge puddles in 1T-TaS_2_; the dI/dV mapping shows little variation in the local electronic structures in Fig. 3c. We note that the thermopower puddles in Fig. 3b exhibits much stronger variation in ΔV, up to 3 mV in 1T-TaS_2_, which requires an additional explanation beyond the conventional charge puddles in the commensurate phase of 1T-TaS_2_.

Considering the formula for the atomic Seebeck effect, ΔV(r)=-S(r)·ΔT(r)^27^, where S(r) is the local Seebeck coefficient, we can interpret the spatially-varying temperature gap ΔT(r) to be another origin for the large-scale ΔV features in Fig. 3b. In the scale of hundreds of nanometers, the local temperature gap ΔT(r) between the tip and sample could be changed by local thermal conductance with a given macroscopic temperature difference (~220 K). In the Mott insulating phase, a small amount of charge carriers drives the heat transport at the tip-sample contact, dominated by local phonon scattering, which we call phonon puddles in this study.

### Microscopic sonar to detect phonon puddles

**Fig. 4:**
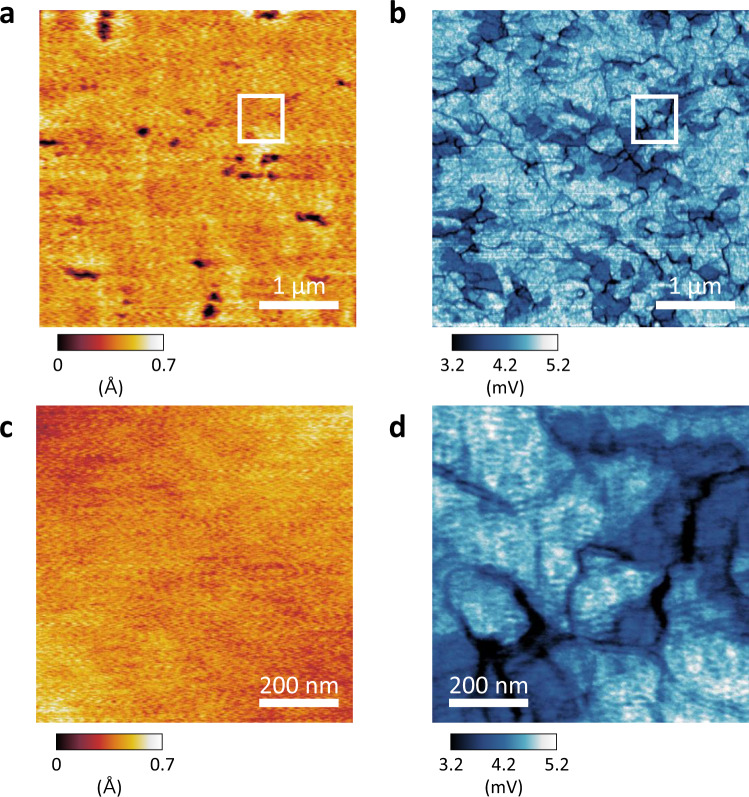
Large-scale imaging of phonon puddles in the commensurate CDW phase of 1T-TaS_2_ by SThEM. **a**, **b** Topography **a** and thermopower **b** images over a distance of 4 μm at T_sample_=78 K. Defect areas (pits and grain boundaries) in **a** exhibit lower thermopower in **b**. The thermopower image shows complex patterns that are not observed in the topography. **c**, **d** Magnified topography **c** and the corresponding thermopower **d** images highlighted by a white rectangle in **a** and **b**. While the flat area in **c** shows no structural disorder, the thermopower mapping in **d** reveals clear patterns with large contrast in ΔV, which originates from phonon puddles and the resulting effective ΔT at the junction between the tip and sample.

Phonon puddles and the resulting spatial distribution of ΔT(r) were clearly observed in the large-scale SThEM mapping of 1T-TaS_2_ as shown in Fig. 4. The topography in Fig. 4a shows several pits and domain boundaries, which are correlated to the darker areas in the simultaneously obtained thermopower mapping image in Fig. 4b.

To clearly demonstrate the exotic phonon puddles on atomically-flat areas, magnified topography and corresponding ΔV mapping images are shown in Fig. 4c, d. Unlike the atomic-scale thermopower from local electronic structures discussed in Figs. 1–3, the thermopower variation in Fig. 4d reflects the spatial distribution of ΔT, which is determined by local phonon scattering or phonon puddles reflecting the subsurface structures of the flat area (Fig. 4c). Our SThEM works as a microscopic sonar to detect below the structure-driven phonon puddles in the thermopower mapping even in the atomically-flat area (Fig. 4c, d).

## Discussion

Layered transition metal dichalcogenides (including 1T-TaS_2_) commonly possess structural disorders, such as stacking faults^30^, grain boundaries, defects (e.g., vacancies and interstitial atoms), and ripples. It has been reported that thermal conductivity via phonon propagation is affected by these structural disorders, which have been used to optimize the thermoelectric figure of merit ZT^40^. The structural disorders in the subsurface structures in Fig. 4c, d affect the phonon puddles, which could be sensitively probed in the Mott insulating phase of 1T-TaS_2_ with SThEM.

In this study, we demonstrate drastic variation in thermopower (4 mV) over an atomic lattice constant (<1 nm) with a broken three-fold rotational symmetry in a strongly correlated electronic system, 1T-TaS_2_, which violates our conventional understanding of macroscopic thermoelectricity. Our first-principles calculations and in-situ STM and tunneling spectroscopy experiments reveal the peculiar role of interlayer coupling and the Mott insulating phase in the novel thermoelectric features. Moreover, phonon puddles, an unprecedented fluctuation in local temperature, were probed with a spatial resolution (nanometer scale) shorter than conventional phonon diffusion lengths (micrometer scale)^41^ in the correlated electronic system.

## Methods

### Sample preparation

The 1T-TaS_2_ sample was purchased from 2D Semiconductors and cleaned by in-situ cleaving the top several layers in an ultra-high vacuum (UHV) chamber (phase transition temperature from 1T to 2H phase is about 250 °C) to avoid possible oxidation or absorption on the surface.

### Scanning thermoelectric microscopy (SThEM) measurement

We used an Omicron VT-SPM system for SThEM with a based pressure of 10^−11^ torr. The STM preamplifier was replaced by a high-impedance electrometer. All SThEM measurements were performed with a platinum-coated metallic cantilever. While the metallic tip was kept at room temperature, the 1T-TaS_2_ sample was heated by a PBN resistive heater or cooled by continuous-flow liquid nitrogen to generate a nominal temperature gradient ΔT at the tip-sample interface. The temperature gradient driven thermoelectric voltage was measured by a high-impedance electrometer in contact-AFM mode with a constant force feedback loop. The current flow in our SThEM is negligible with a high impedance (200 TΩ, Keithley 6517B) electrometer, which excludes the Joule heating effect at the junction. Moreover, the long retention time in our SThEM (5 ms) neglects the transient current during the heat transfer process at the junction within 1 ns time scale. The surface height variation and thermoelectric voltage mapping were obtained simultaneously, with a weak contact force range of 10^−8^ to 10^−11^ N.

The averaged thermoelectric voltage (Fig. 1b) in a certain region was taken as the central value of the Gaussian distribution in each ΔT-induced thermoelectric voltage image. The experimental setup-induced voltage at ΔT = 0 K was subtracted for better clarify.

### Measurement of phonon puddles by SThEM

Temperature is a value in statistics with numerous particles, but it can vary abruptly when phonons scatter at atomic-scale disorders. The varying quantity at the atomic scale in our SThEM study is “the temperature drop at the interface (written by ΔT(r) in the manuscript)”.

To clarify the measurement of ΔT(r) variation at the atomic scale, we demonstrate a case of T_sample_ = 80 K and T_tip_ = 300 K in SThEM. The two temperatures appear far from the junction (i.e., the interface between the tip and sample) as heat reservoir. Once the tip and sample (1T-TaS_2_ in our study) make a contact, heat transfer occurs and temperature gradients (across the heat reservoir and the junction) are formed in the bulk tip (Δ*T*_*tip*_*(r)*) and sample (Δ*T*_*sample*_*(r)*), described by a formula below:

1 $${\Delta T}_{{tip}}(r)+{\Delta T}_{{interface}}(r)+{\Delta T}_{{sample}}(r)=220\,K$$

where ${\Delta T}_{{interface}}(r)$ is the temperature drop at the interface between the tip and sample. “Δ*T(r)*” in our manuscript indicates ${\Delta T}_{{interface}}(r)+{\Delta T}_{{sample}}(r)$ in the formula. Among the three terms, Δ*T*_*tip*_*(r)* barely changes because we use the same geometry tip for the scanning. Since the eigenstates of phonons are different in the tip and sample (1T-TaS_2_), the interfacial phonon scattering produces a finite thermal conductance and ${\Delta T}_{{interface}}(r)$.

Although (macroscopic) temperature itself cannot vary at the atomic scale, the three terms in the above formula vary at the atomic scale at the junction (interface) due to local (spatially-varying) phonon scattering. Local atomic arrangements, symmetry variation, or surrounding defects can generate spatially-varying phonon scattering or local heat conductance, which perturbs the three temperature gradients (Δ*T*_*tip*_*(r)*, Δ*T*_*interface*_*(r)*, and Δ*T*_*sample*_*(r)*) in the above formula.

The spatially-varying measured voltage in our SThEM can be expressed by a formula below:

2 $${\Delta T}_{{tip}}\left(r\right)\bullet {S}_{{tip}}\left(r\right)+{\Delta T}_{{interface}}\left(r\right)\bullet {S}_{{interface}}\left(r\right)+{\Delta T}_{{sample}}\left(r\right)\bullet {S}_{{sample}}\left(r\right)=\triangle V\left(r\right)$$

where Seebeck coefficients, S(r), of the tip, interface, and sample are multiplied with corresponding temperature gaps, ΔT.

### Scanning tunneling microscopy (STM) and spectroscopy (STS)

All STM measurements were performed with a chemically etched tungsten tip using an Omicron VT-SPM system under a base pressure of 10^−11^ torr. For the low temperature measurement, the sample was cooled by a continuous liquid nitrogen cooling system. The STS was conducted on different CDW phases of the 1T-TaS_2_ sample, using a Stanford SR830 lock-in amplifier with a modulation voltage of 0.04 V and a frequency of 911.1 Hz.

### First-principles calculations and scanning Seebeck simulation

The Vienna Ab Initio Simulation package (VASP)^42^ was used for density functional theory (DFT) calculations with the Perdew-Burke-Ernzerhof exchange-correlation functional (PBE)^43^. We used the energy cutoff of plane waves of 800 eV for a proper representation of wavefunction decaying in a vacuum. The van der Waals (vdW) interaction between interlayers was corrected with the DFT-D3 method^44^. We also used the +*U* correction for an accurate description of the strong-correlated electronic features; the *U* parameter was 2.27 eV for Ta, which was calculated from the linear-response theory for pure 1T-TaS_2_^45^. The spin-orbit coupling was taken into account. The Γ-centered 7 × 7 × 1 Brillouin zone sampling was used for all calculations. The total energy precision and ionic relaxation force criteria were 0.1 meV and 0.01 eV/Å, respectively. The local density of states (LDOS) was calculated using the linear tetrahedron method^46^.

The scanning Seebeck simulation^27^ was performed at the vdW equilibrium height *z*(r) between the tip (assumed to be a single tungsten atom) and sample, which can be called the van der Waals topography. The vdW energy was evaluated from the 12-6 Lennard-Jones (L-J) potential

3 $${E}_{{{{{{\rm{vdW}}}}}}}\left({{{{{{\boldsymbol{r}}}}}}}_{{{{{{\boldsymbol{i}}}}}}}\right)=\mathop {\sum} \limits _{{{{{{\boldsymbol{j}}}}}}}4{\varepsilon }_{{ij}}\left[{\left(\frac{{\sigma }_{{ij}}}{\left|{{{{{{\boldsymbol{r}}}}}}}_{{{{{{\boldsymbol{i}}}}}}}{{{{{\boldsymbol{-}}}}}}{{{{{{\boldsymbol{r}}}}}}}_{{{{{{\boldsymbol{j}}}}}}}\right|}\right)}^{12}-{\left(\frac{{\sigma }_{{ij}}}{\left|{{{{{{\boldsymbol{r}}}}}}}_{{{{{{\boldsymbol{i}}}}}}}{{{{{\boldsymbol{-}}}}}}{{{{{{\boldsymbol{r}}}}}}}_{{{{{{\boldsymbol{j}}}}}}}\right|}\right)}^{6}\right].$$

where ${{{{{{\boldsymbol{r}}}}}}}_{{{{{{\boldsymbol{i}}}}}}}$and ${{{{{{\boldsymbol{r}}}}}}}_{{{{{{\boldsymbol{j}}}}}}}$ are the atomic positions for the tip and sample, respectively. The L-J potential parameters ${\varepsilon }_{{ij}}$ and ${\sigma }_{{ij}}$ are defined as ${\varepsilon }_{{ij}}=\sqrt{{\varepsilon }_{{ii}}{\varepsilon }_{{jj}}}$ and ${\sigma }_{{ij}}=\left({\sigma }_{{ii}}+{\sigma }_{{jj}}\right)/2$. The L-J potential is summed for a distance within 15 Å from the tip. Each parameter used in the calculation is listed in Supplementary Fig. 12.

The Landauer formula based on the Tersoff-Hamann approximation was used for the thermoelectric transport property in a coherent regime^27^.

4 $$S=-\frac{1}{\left|e\right|T}\frac{\int N\left(E\right)\left(-\frac{\partial f}{\partial E}\right)\left(E-{E}_{{{{{{\rm{F}}}}}}}\right){dE}}{\int N\left(E\right)\left(-\frac{\partial f}{\partial E}\right){dE}}$$

where *S* is the Seebeck coefficient, *T* is the temperature, *N(E)* is the LDOS at an energy of *E*, $\partial f/\partial E$ is the energy derivative of the Fermi-Dirac distribution, and *E*_F_ is the Fermi level. An energy grid of 10 meV was used, and the convergence of the energy window for numerical integration was fully confirmed.

### Rough estimate of ΔT for phonon puddles

Macroscopic area of the sample is not heated up to room temperature as the sample keeps its commensurate CDW phase in our experiment (which indicates the sample temperature is below T=220 K, commensurate CDW transition temperature).

The surface temperature or thermal conductance should change by the heat flow. However, we only observe the variation of temperature gap (ΔT(r)) in the sample parts, and thus, the absolute temperature of the surface (modified by the contact) cannot be obtained in this study.

A rough estimation of ΔT (due to the heat flow) could be made by using the averaged value of S (~200 μV/K) and ΔV over the area (e.g., Figs. 2d and  4). The averaged ΔV is 4 mV, which implies that the averaged ΔT = 20 K over the area. The spatial distribution of ΔT is demonstrated in Supplementary Fig. 11. The phonon puddles (local heat conductance) produce a variation of ΔT up to 20 K in Supplementary Fig. 11. We believe that the temperature gap variation is enough to be observed as phonon puddles.

## Supplementary information

Supplementary Information

## Data Availability

All data are available in the main text or the supplementary information. The data that support the findings of this study are available from the corresponding author upon reasonable request.
