# Supplementary material for: Atomic-scale thermopower in charge density wave states
Source: Nat Commun. 2022 Aug 3;13:4516. doi: 10.1038/s41467-022-32226-y (PMC9349257; doi:10.1038/s41467-022-32226-y)
Supplement: Supplementary file 1 — Supplementary Information [file 41467_2022_32226_MOESM1_ESM.pdf]

Supplementary Information for

Atomic-scale thermopower in charge density wave states

Dohyun Kim[1†], Eui Cheol Shin[2†], Yongjoon Lee[2], Young Hee Lee[1,3], Mali Zhao[4]*, Yong-Hyun Kim[2]*, Heejun Yang[2]*

[1]Department of Energy Science, Sungkyunkwan University, Suwon 16419, Korea

[2]Department of Physics, Korea Advanced Institute of Science and Technology (KAIST), Daejeon 34141, Korea

[3]Center for Integrated Nanostructure Physics (CINAP), Institute for Basic Science, Suwon 16419, Korea

[4]Interdisciplinary Materials Research Center, College of Materials Science and Engineering, Tongji University, Shanghai 201804, People's Republic of China

Correspondence authors: Mali Zhao (mali_zhao@hotmail.com), Yong-Hyun Kim (yong.hyun.kim@kaist.ac.kr), and Heejun Yang (h.yang@kaist.ac.kr)

[†]These authors contributed equally to this work.

This Supplementary Information includes:

Supplementary Fig. 1 to 12
Supplementary References

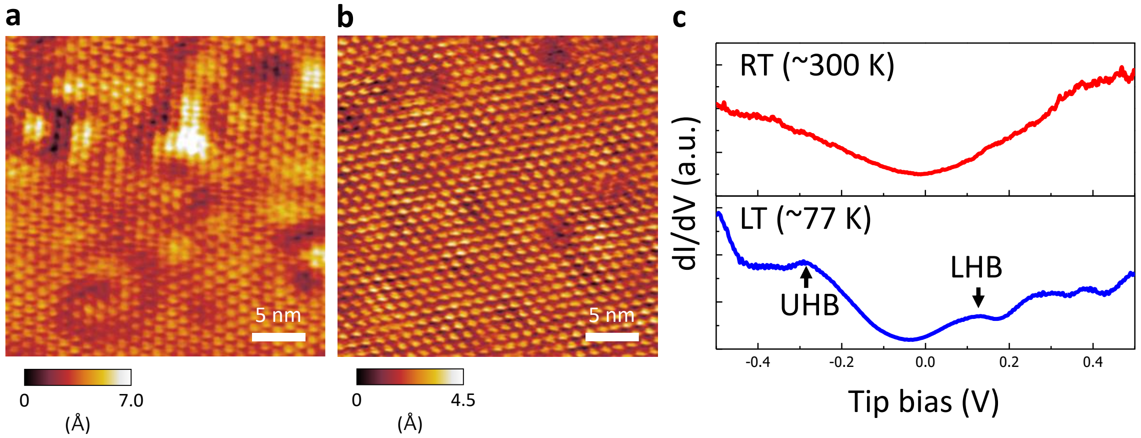

Supplementary Fig. 1: STM topography image and STS of the 1T-TaS_2 sample. **a**, STM topography image of TaS_2 in the nearly commensurate (NC)-CDW phase at room temperature. Tunneling condition: V_s = -300 mV, I = 0.3 nA. **b**, STM topography image of commensurate-CDW TaS_2 at ~ 77 K (liquid nitrogen temperature). Tunneling condition: V_s = 500 mV, I = 0.3 nA. **c**, Temperature dependent dI/dV spectra vs tip bias on 1T-TaS_2. At room temperature, TaS_2 shows a metallic phase without a band gap (red curve in upper panel); at 77 K, a Mott-insulating gap of 0.42 eV is open (blue curve in lower panel). The black arrows point to the upper (UHB) and lower Hubbard band (LHB) at -0.29 eV and +0.13 eV, respectively, indicating the dominant charge carrier is a hole.

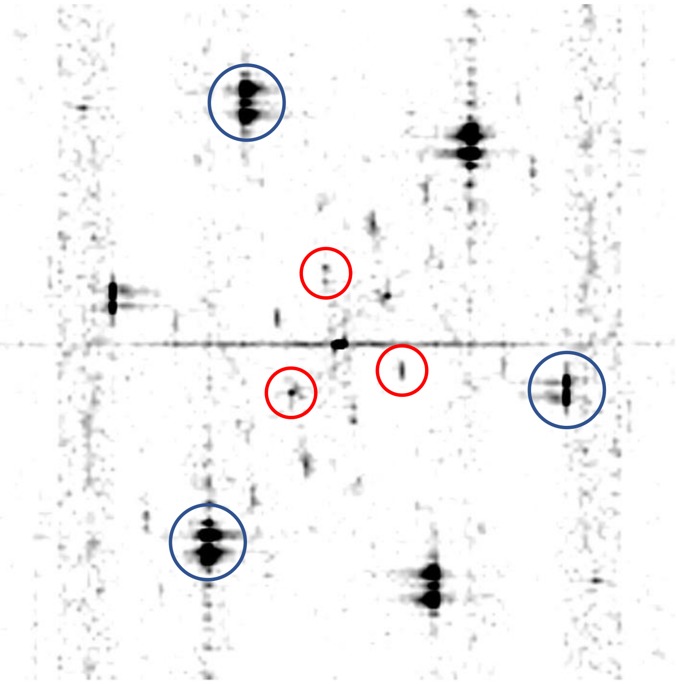

Supplementary Fig. 2: The FFT of the topography in Fig. 1c. Bragg peaks are indicated by blue circles, and the CDW superlattice peaks are indicated by red circles.

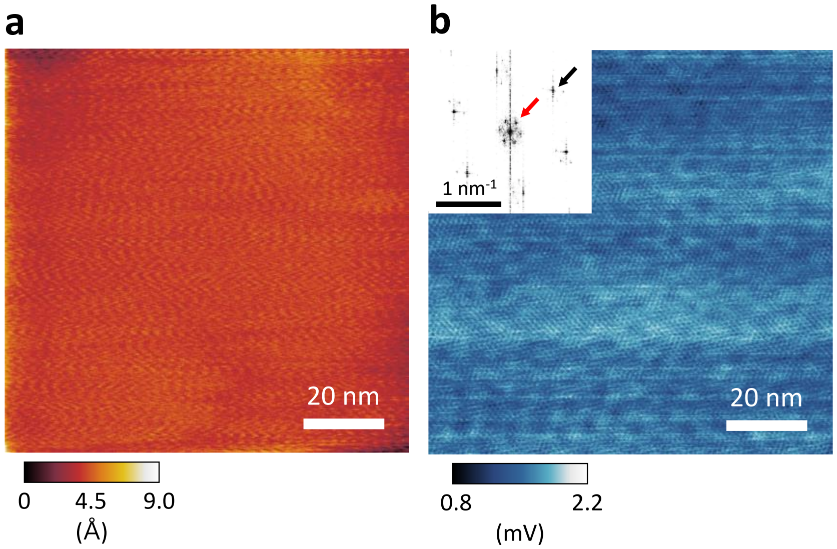

Supplementary Fig. 3: Topography and thermoelectric voltage images of 1T-TaS_2 in the NC-CDW phase by SThEM. a, Topography image of the 1T-TaS_2 nearly-commensurate CDW phase at 320 K (ΔT= 20 K). **b**, The simultaneously obtained thermoelectric voltage image of 1T-TaS_2. The inset panel is the FFT pattern of the thermoelectric voltage image. The peaks marked by the black arrow indicate the hexagonal superstructure of SoD. The peaks near the gamma point (marked by the red arrow) clearly indicate the hexagonal domain-like structure with a period of ~ 7 nm, containing a commensurate CDW domain and a boundary[29]. The rotation angle between the domain-like structure and the superlattice of SoD is 10° (±2°).

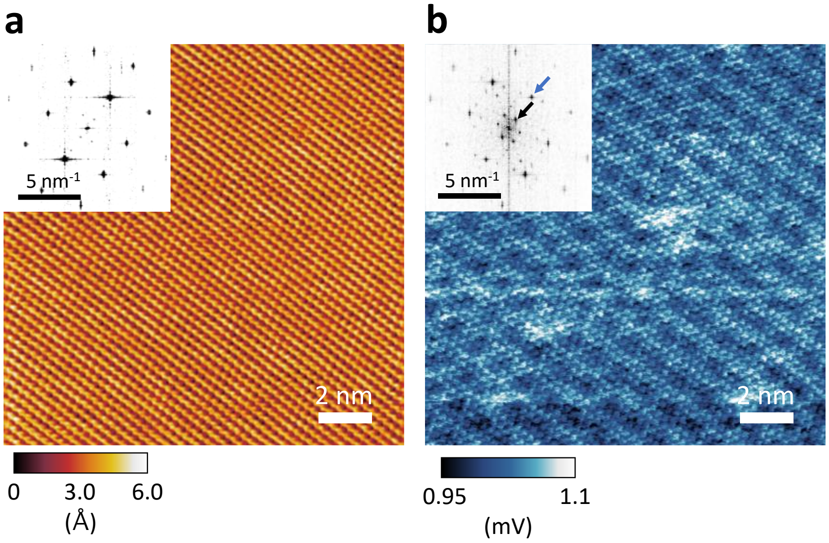

Supplementary Fig. 4: Topography and thermoelectric voltage images of 1T-TaS_2 in the incommensurate (IC)-CDW phase by SThEM. a, Topography image of the 1T-TaS_2 sample at 400 K (ΔT= 100 K). The inset panel is the FFT pattern of the topography image. **b**, Simultaneously obtained thermoelectric voltage image of 1T-TaS_2 in the IC-CDW phase. The inset panel is the FFT pattern of the thermoelectric voltage image. The black arrow indicates the superlattice of SoD, the blue arrow points to the atomic lattice. The SoD superlattice is incommensurate and aligned with the atomic lattice.

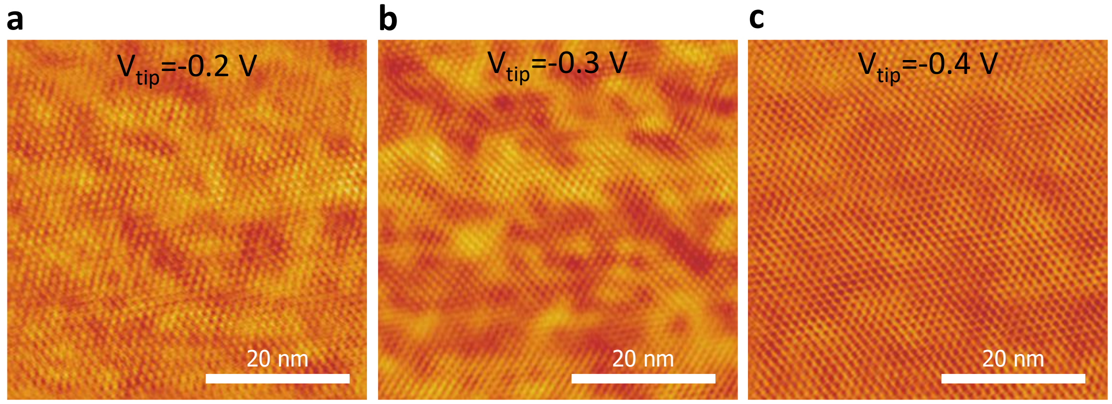

Supplementary Fig. 5: A series of dI/dV mappings of 1T-TaS_2 in the commensurate CDW phase at different tip bias voltages. The spatial fluctuations in the dI/dV mappings (proportional to the DOS near the Fermi level) suggest the charge inhomogeneity in TaS_2 caused by defects in the surface (or subsurface) or local strain. Among them, the dI/dV mapping at the tip bias of -0.3 V indicates the strong spatial inhomogeneity of the UHB edge.

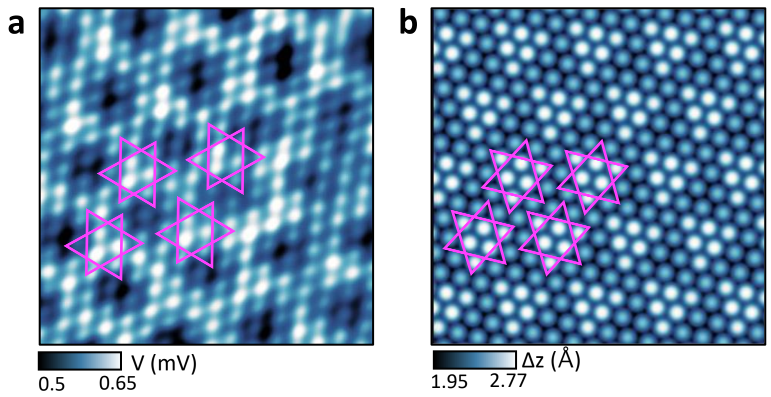

Supplementary Fig. 6: Resemblance between thermoelectric image and van der Waals topography. a,**b**, Experimental thermoelectric image (**a**) of the NCCDW phase and theoretical vdW topography (**b**) of 1T-TaS_2. This clearly shows the significant bright protrusions of sulfur atoms. The Star-of-David is marked. In the NCCDW phase at room temperature, the spatial variation of the Seebeck coefficient is very small due to the metallic electronic structure. Therefore, the temperature gap according to the difference in vdW interaction depending on position may have a major effect on thermoelectric imaging.

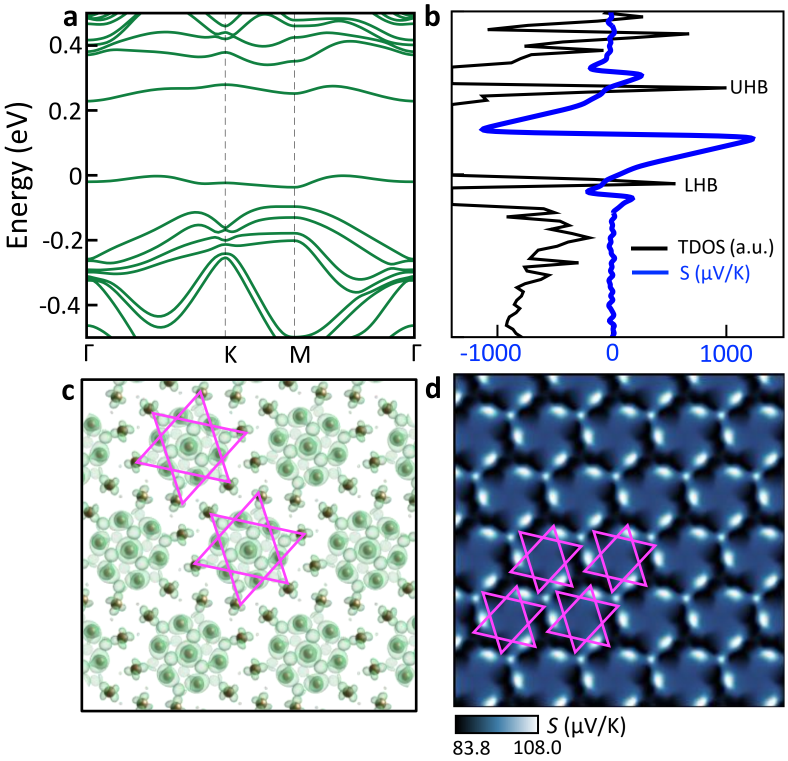

Supplementary Fig. 7: First-principles scanning Seebeck simulation of single layer 1T-TaS_2. **a**,**b**, Electronic band structure (**a**) and the corresponding density of states (**b**) of CDW monolayer 1T-TaS_2. The upper Hubbard band is at +0.22 eV, and the Mott gap is 0.22 eV. The calculated Seebeck coefficient, which is critically changed depending on the Fermi energy position, is also displayed. **c**, CDW in single-layer 1T-TaS_2 at valence band maximum (VBM). The Star-of-David is seen with a $\sqrt{\mathbf{13}} \times \sqrt{\mathbf{13}}$ supercell, marked with a magenta star. The sulfur atoms are not presented for clarity. **d**, Scanning Seebeck simulation image at VBM. Due to the high charge density population around the SoD center, the magnitude of the Seebeck coefficient is low at the center; on the other hand, the Seebeck coefficient is relatively large between SoDs because of the sparse charge density.

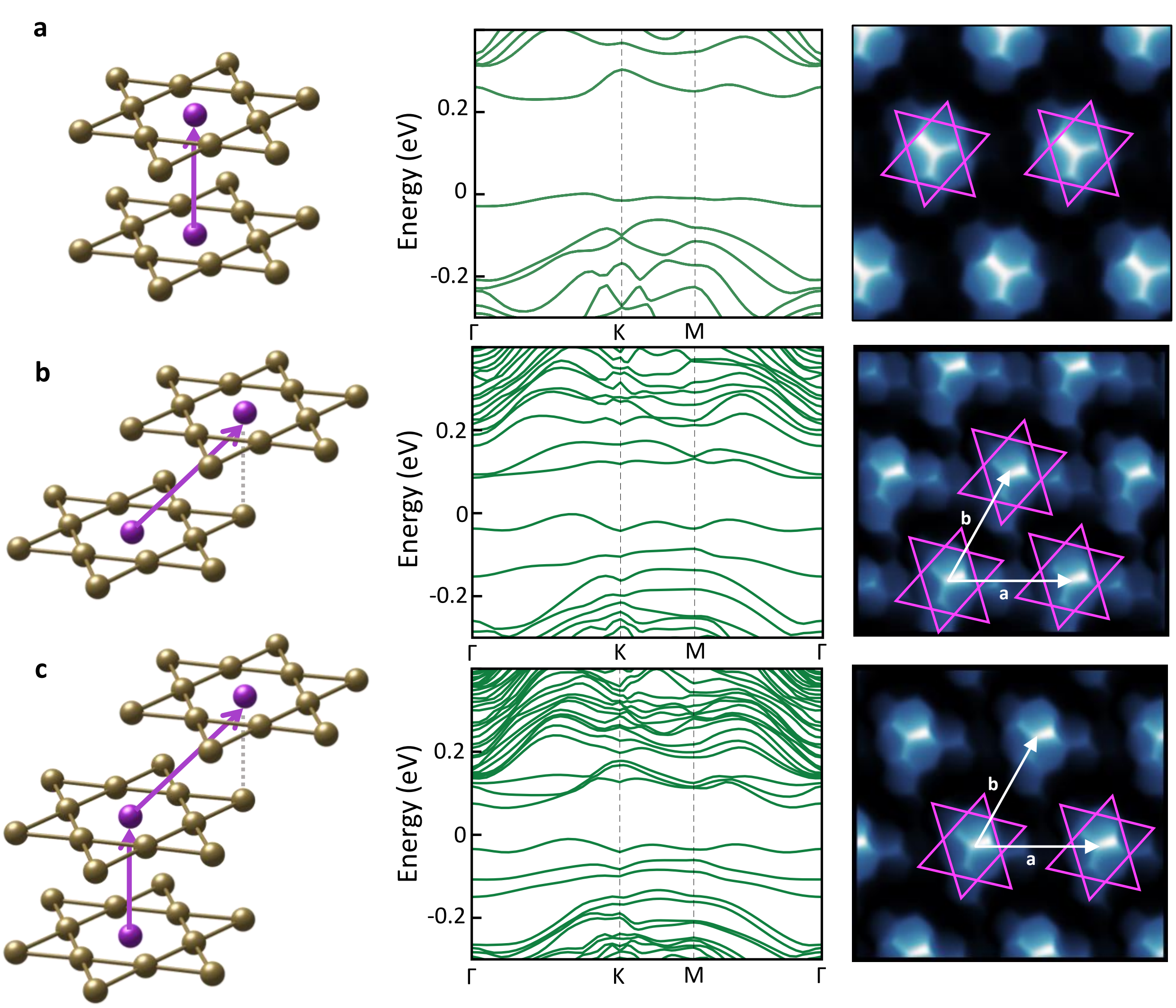

Supplementary Fig. 8: First-principles scanning Seebeck simulation of multilayer 1T-TaS_2. (Left) Atomic structure, (Middle) band structure, and (Right) charge density image of multilayer 1T-TaS_2 with **a**,**b**,**c**, *A* stacking (**a**), *L* stacking (**b**), and *AL* stacking (**c**). The center Ta atom of the Star-of-David is highlighted in purple. The charge densities in the *L* and *AL* stackings show a broken three-fold symmetry character. The a and b are lattice vectors.

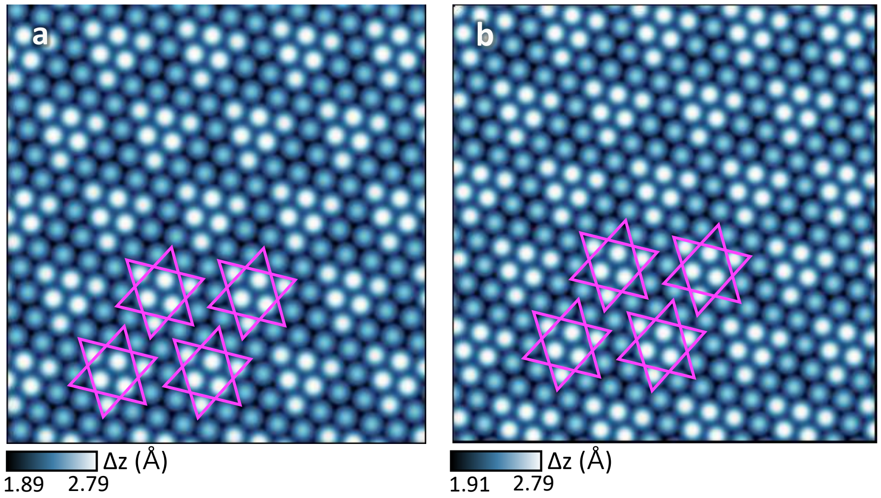

Supplementary Fig. 9: vdW topography of bilayer 1T-TaS_2. a,**b**, *A* (**a**) and *L* (**b**) stacking. The three-fold symmetry is maintained around the top surface Star-of-David. Except for a very small difference of 0.02 Å in the minimum height, the overall profile is almost identical.

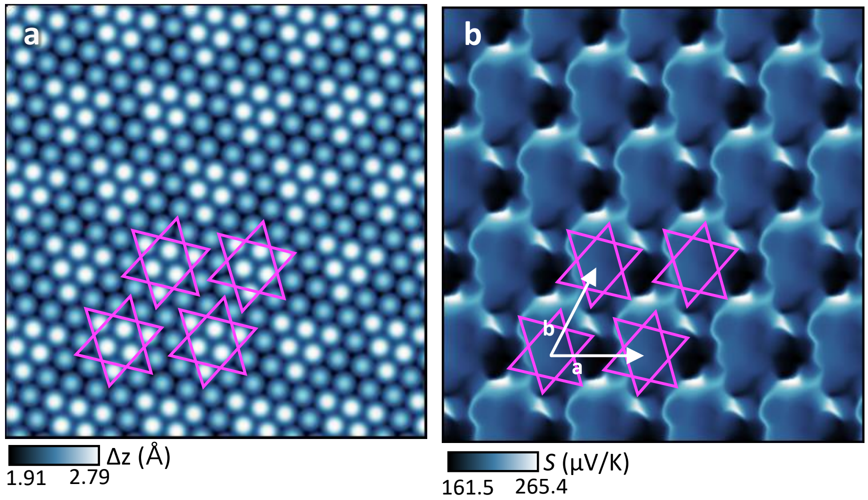

Supplementary Fig. 10: First-principles scanning Seebeck simulation of multilayer 1T-TaS_2 in *AL* stacking. a,**b**, vdW topography (**a**) and scanning Seebeck simulation image (**b**) of *AL* stacking. The vdW topography is almost identical to those in Fig. S8. The scanning Seebeck image is also very similar to the image for *L* stacking in Fig. 2d. The a and b are lattice vectors.

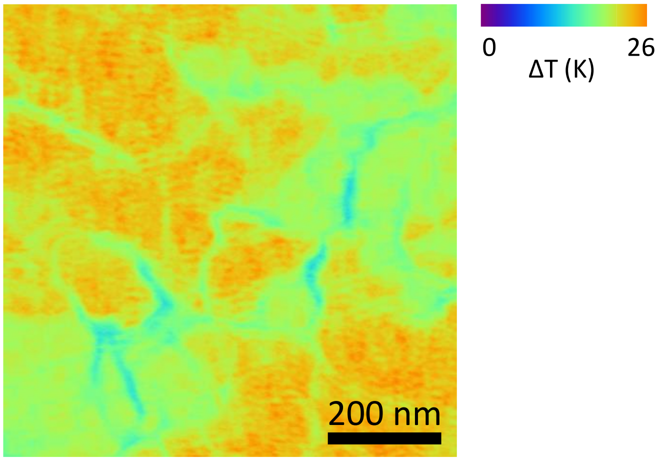

Supplementary Fig. 11: ΔT distribution from Fig. 4d. A rough estimation of ΔT (due to the heat flow) could be made by using the averaged value of S (~200 μV/K) and ΔV over the area shown in Figs. 2d and Fig. 4. The averaged ΔV is 4 mV, which implies that the averaged ΔT=20 K over the area.

Supplementary Fig. 12: Lennard-Jones parameters used in the construction of vdW topography.

i	ε_{ii} (meV)	σ_{ii} (Å)
Ta[1]	980	2.68
W[1]	1060	2.56
S[2]	20.0	3.13

Supplementary References

1. Zhen, S. & Davies, G. J. Calculation of the Lennard-Jones n-m potential energy parameters for metals. *Phys. Status Solidi A, Appl. Res.* **78**, 595–605.
2. Liang, T., Phillpot, S. R. & Sinnott, S. B. Erratum: Parametrization of a reactive many-body potential for Mo--S systems [*Phys. Rev. B* **79**, 245110 (2009)]. *Phys. Rev. B* **85**, 199903 (2012).
